# Dual-modal photoacoustic and magnetic resonance tracking of tendon stem cells with PLGA/iron oxide microparticles in vitro

**DOI:** 10.1371/journal.pone.0193362

**Published:** 2018-04-02

**Authors:** Man Lu, Xueqing Cheng, Jingzhen Jiang, TingTing Li, Zhenqi Zhang, Chialing Tsauo, Yin Liu, Zhigang Wang

**Affiliations:** 1 Chongqing Key laboratory of Ultrasound Molecular Imaging, Second Affiliated Hospital of Chongqing Medical University, Chongqing, China; 2 Ultrasound Medical Center, Sichuan Cancer Hospital Institute, Sichuan Cancer Center, School of Medicine, University of Electronic Science and Technology of China, Chengdu, China; 3 North Sichuan Medical College, Nanchong, China; 4 Department of Pharmacology, West China School of Preclinical and Forensic Medicine, Sichuan University, Chengdu, Sichuan Province, China; 5 Department of Anesthesiology, Sichuan Cancer Hospital & Institute, Sichuan Cancer Center, University of Electronic Science and Technology of China, Chengdu, Sichuan Province, China; Brandeis University, UNITED STATES

## Abstract

Reliable cell tracking is essential to understand the fate of stem cells following implantation, and thus promote the clinical application of stem cell therapy. Dual or multiple modal imaging modalities mediated by different types of multifunctional contrast agent are generally needed for efficient cell tracking. Here, we created a new contrast agent—PLGA/iron oxide microparticles (PLGA/IO MPs) and characterized the morphology, structure and function of enhancing both photoacoustic (PA) and magnetic resonance imaging (MRI). Both PA and MRI signal increased with increased Fe concentration of PLGA/IO MPs. Fluorescent staining, Prussian blue staining and transmission electron microscope (TEM) certified that PLGA/IO MPs were successfully encapsulated in the labeled TSCs. The established PLGA/IO MPs demonstrated superior ability of dual-modal PA/MRI tracking of TSCs without cytotoxicity at relatively lower Fe concentrations (50, 100 and 200 μg/mL). The optimal Fe concentration of PLGA/IO MPs was determined to be 100 μg/mL, thus laying a foundation for the further study of dual-modal PA/MRI tracking of TSCs in vivo and promoting the repair of injured tendon.

## Introduction

Stem cells are characterized by the multi-differential potentialities and the capacity of self-renewal, which has been applied in disease therapy, especially in regenerative medicine[1]. Tendon stem cells (TSCs) has shown great potential as a new strategy to improve repair of tendon injury since Bi et al. isolated tendon stem/progenitor cells from mouse and human tendon and proved its’ ability of regenerating tendon-like tissues[2]. In order to promote the clinical application of TSCs, there is a growing need to track TSCs for a better understanding of migration, distribution and engraftment following transplantation.

There are various kinds of imaging modalities applied in cell tracking including Magnetic Resonance Imaging (MRI), Optical Imaging, Radioactive Imaging (SPECT and PET), Ultrasound (US) and Photoacoustic Imaging (PA)[3]. Optical Imaging has high sensitivity and high resolution, but the clinical applicability is limited due to shallow penetration depth and phototoxicity[4]. The insufficiencies of radioactive imaging are low spatial resolution, cytotoxicity and short half-life of the radionuclide[5]. Conversely, while MRI has the advantages of providing anatomical information together with a good spatial and temporal resolution that made it a widely used imaging modality for cell tracking, it has lower sensitivity when compared to photoacoustic (PA) or fluorescence methods[6]. PA is a compound imaging technology that includes optical imaging and US imaging based on photoacoustic effect, which allows visualization of morphological, functional, and molecular properties from organelles to organ[7]. The main application of PA in biomedicine is reflecting the oxygen saturation of tissues, tumor/ inflammation angiogenesis, early cancer detection and treatment, cell tracking and so on[7–9]. However, application of PA in cell tracking is still in its’ early days as compared to MRI beyond a proof of principle.

Due to intrinsic limitations and weaknesses of each imaging technique, dual or multiple modal imaging modalities by using multifunctional contrast agent are generally needed for the efficient tracking of stem cell. One of the earliest multifunctional nanoparticles integrated the near-infrared dye for fluorescence imaging with the iron oxide nanoparticles for MRI, thus providing the dual-modal fluorescence/MRI method to visualize brain tumor preoperative and intraoperative which cannot be realized by individual imaging[10]. In addition, other imaging techniques have also been combined for multimodal tracking of stromal stem cells (MSCs) based on constructed multifunctional contrast agents, such as fluorescence/PA imaging, MRI/PA imaging, and US/PA imaging[11, 12]. These multimodal nanoprobes were usually synthesized by an “all in one” concept, which means integrating different functional blocks in one particle[13]. This may result in a complicated structure and increased cytotoxicity. For cell tracking, we need higher biosafety “one for all” nanoprobes, which involves intrinsic properties of the element in a single particle[13]. These “one for all” methods include gold-based nanoparticles for X-rays/PA imaging[14], copper-based nanoparticles for MRI/PA imaging[15], tungsten-based nanoparticles for CT/PA imaging et.al[16]. However, only a few of these methods had been applied in cell labeling and monitoring. In the case of cell tracking, enhancement reagents are selected with little cytotoxicity, minimal effects on cell’s biological functions, and the availability of a noninvasive method.

Iron oxide microparticles (IO MPs) especially superparamagnetic iron oxide nanoparticles (SPIOs) have been extensively explored for different biomedical application due to its’ benefits of good biocompatibility and unique magnetic property. They were initially developed and FDA approved as MRI negative contrast agents and have been proposed for cancer imaging, imaging-guided cancer therapy such as photothermal therapy[17, 18], photodynamic therapy[19] and hyperthermia[20] and cell tracking[21, 22]. When used for stem cell labeling, IO MPs are often coated with polymers as they can provide both colloidal stability and sufficient protection of the iron oxide core at physiological pH.

In our current study, we used organic poly (lactide-co-glycolide) (PLGA) embedded with inorganic iron oxide (IO) nanoparticles to prepare the PLGA/IO microparticles (PLGA/IO MPs). These prepared PLGA/IO MPs demonstrated the feasibility of labeling TSCs via dual-model MRI/PA imaging. We assessed the sensitivity as well as quantification of MRI and PA imaging for tracking TSCs in vitro. The optical concentration of PLGA/IO MPs for labeling, pertaining to the cytotoxicity, and labeling efficiency of MRI and PA imaging was determined in this work. The foundation for in vivo MRI/PA dual-modal tracking of TSCs in the rat rotator cuff injury model will be laid.

## Materials and methods

### The isolation, culture and identification of rat TSCs

This animal study was carried out in accordance with the recommendations in the Guide for the Care and Use of Laboratory Animals of the National Institutes of Health. The protocol was approved by the animal protection and care committee of Second Affiliated Hospital of Chongqing Medical University (No.398, 2016). All surgery was performed under sodium pentobarbital anesthesia, and all efforts were made to minimize suffering. All animals were sacrificed by air embolism. TSCs were harvested from 6 ~ 8 weeks old male SD rats based on the previous protocol described by Bi et.al[2]. Following anesthesia, the bilateral achilles tendons were acquired and cut into small pieces (1 mm×1 mm), then digested with 3 mg/mL collagenase type I Sigma, C0130-1G and 4 mg/mL dispase (Sigma, D4693-1G) in PBS for 2 h at 37°C. The isolated cell suspensions were primary cultured and subcultured in DMEM (Gibco), supplemented with 10% (v/v) FBS (Hyclone) and 1% (v/v) penicillin/ streptomycin (Beyotime) at 37°C, 5% CO_2_. Cells were cultured to 70~80% confluence before passaging. All experiments were performed using P3 ~P7 TSCs. Before cell experiments, TSCs were detached with Trypsin 0.05%-EDTA 0.53 mM (Gibco).

For identification of TSCs, the colony formation of P1 TSCs and the proliferation of TSCs were observed. Stem cell markers SSEA4, Nucleostemin, CD90 and CD44 were examined using immunocytochemistry. The TSCs were fixed with 4% paraformaldehyde in PBS for 30 min at room temperature, permeabilized with 0.25% Triton X-100 in PBS and blocked with 1% BSA for 30 min at room temperature. The cells were then reacted with primary anti-SSEA4 antibody (1:100; Abcam, ab16287), anti-Nucleostemin antibody (1:100; Abcam, ab70346) anti-CD44 antibody (1:50; Abcam, ab194987) and anti-CD90 antibody (1:100; Abcam, ab92574) for overnight at 4°C. After washing the cells with PBS, the secondary antibody was applied for 2 h at room temperature. DAPI was used to stain the cell nuclei (blue) at a concentration of 1.43 μM. The staining results were observed with confocal laser scanning microscope.

### Preparation of PLGA/IO MPs

PLGA/IO MPs were prepared using double emulsion method. Briefly, 200 uL Fe_3_O_4_ NPs (10 nm, 25 mg/mL, Ocean Nanotech, AR) coated with oleic acid, 200 uL deionized water (H_2_O) were mixed with 30 mg PLGA (lactide: glycolide = 50:50, MW = 10000Da, Daigang Biomaterial Co., Ltd, Jinan, ShanDong) in 1 mL chloroform, then the solution was sonicated for 1 min at 125w using acoustic vibration (VCY-500, Shanghai, China) to achieve an emulsion in a 50 mL beaker. Subsequently, 10 mL 4% PVA solution was added and dispersed by a high-shear dispersion homogenizer at B level (HENC) for 2 min to form the second emulsion. The homogenized mixture was then stirred for 4 h in a chemical fume hood at room temperature to evaporate chloroform. Finally, particles were isolated by centrifugation at 6,000 rpm for 5 minutes, washed twice with deionized water, and then redispersed in 1.5 mL deionized water and stored at 4°C for standby application. The procedure of preparing PLGA/IO MPs was repeated for at least three times to make the same particles.

PLGA/H_2_O MPs were prepared by using equal volume of deionized water for substitution of Fe_3_O_4_ NPs using the same protocol mentioned above. The PLGA/H_2_O MPs were utilized as a control for in vitro PA/MRI imaging experiment.

### Characterization of PLGA/IO MPs

The shape and distribution of PLGA/IO MPs suspension was observed instantly by an inverted fluorescence microscope. The morphology and size distribution of the PLGA/IO MPs were estimated by a transmission electron microscope (TEM, JEM-2100F). The zeta potentials, hydrodynamic diameter and polydispersity of particles were collected by Malvern Zetasizer Nano ZS90 in water by averaging 3 runs. To determine the aggregation and stability of PLGA/IO MPs, the prepared particles were observed by microscope after being stored for a month. Iron content of particles was determined using ICP (HORIBA JOBINYVON, model: Activa) after digestion in 70% nitric acid.

For PA imaging, the PLGA/IO MPs particles were diluted to different concentrations (0, 25, 50, 100, 200, and 400 μg Fe/mL) in water and then pipetted into 3% agarose-gel-made holes. The Visual Sonics Vevo LAZR-2100 high-frequency photoacoustic system was used in this study. Initially, B-mode was applied to locate the holes added with samples. Then PA-mode was switched on using a 700 nm wavelength for irradiation, the PA-mode images were acquired and the average PA signal value of each group was measured by drawing the outline of the ROI.

For MRI, the particles were mixed with 1% agarose gel solution at different concentrations (0, 25, 50, 100, 200, and 400 μg Fe/mL) in 1.5 mL EP tube. After 12 hours, MRI samples were imaged by a 3.0 T whole body MRI (Philips Chielva) with a head coil using the T2* sequence (TR = 500 ms, TE = 7.9–63.2 ms, 8 echo, FOV = 80 mm, matrix = 128 × 128, slice thickness = 2.5 mm, flip angle 90°). The signal value of each group was measured by the imaging analysis software matched with the 3.0 T MRI apparatus.

### TSCs labeling with PLGA/IO MPs

For the purpose of TSCs labeling, PLGA/IO MPs were incubated with 0.01% Poly-L-lysine (PLL) for 40 minutes at room temperature in PBS. At 80% confluency, TSCs were incubated with culture medium containing the PLGA/IO particles at the iron concentration of 100 μg Fe/mL for 12 h. Then the old culture medium was removed. Labeled cells were washed 3 times with PBS and permitted to recover in fresh media (10% FBS).

TSCs were labeled with the fluorescent PLGA/IO MPs (incorporating DiI during the preparation of particles) to facilitate the identification of particles internalized within TSCs. At 4 h after labeling, TSCs were fixed with 4% paraformaldehyde, and then stained with fluorescent dyes (with DiO staining plasma membrane and DAPI staining nucleus) and Prussian blue (Beijing Leagene Biotech. Co., Ltd.) according to the manufacturer’s respectively instructions. Unlabeled TSCs were also stained with fluorescent dyes and Prussian blue to be utilized as a control. The inverted fluorescence microscope was applied to observe these two kinds of staining of TSCs. In order to characterize the location of internalized PLGA/IO MPs, 10^6^ labeled TSCs were collected and prepared for TEM scanning according to the previously described method[22].

### Cytotoxicity of PLGA/IO MPs on MSCs

TSCs were seeded in 96-well plate at a density of 5 ×10^4^ per well at 37°C, 5% CO_2_ for 24 h. PLL coated PLGA/IO MPs dispersed in medium at different concentration (0, 25, 50, 100, 200, and 400 μg Fe/mL) were added to the wells and incubated for 24 hours at 37°C. The cells was then washed by PBS twice, and Cell Counting Kit-8 reagent (Dojindo, Lot.JM754) were added into the wells (10 uL per well) and incubated for 2 h. Subsequently, optical density of each well was detected using microplate reader at 450 nm of wavelength. The unlabeled TSCs were considered as control group. The viability of TSCs was then calculated.

### Dual-modal PA/MRI imaging of TSCs labeled with PLGA/IO MPs

TSCs were seeded in 6-well plate at a density of 5 ×10^5^ per well at 37°C, 5% CO_2_. At 80% confluency, TSCs were incubated with culture medium containing the PLL coated PLGA/IO particles at different iron concentration (25, 50, 100 and 200 μg Fe/mL) for 12 h. The unlabeled TSCs were considered as control. The cells were then permitted to recover in fresh media (10% FBS) for 4 h before being collected with 1X trypsin. Finally, the collected cells (2 wells for each group) were counted and homogenously mixed with 500 uL heated 1% agarose solution in 1.5 mL EP tube.

During PA imaging, 100 μL TSCs mixed with 1% heated agarose solution were pipetted from 1.5 mL EP tube of each group and then instantly added into 3% agarose gel model with holes. After cooling and setting, the samples were imaged by the photoacoustic system with the method mentioned above. In turn, during MRI the samples were placed in 1.5 mL EP tubes and each group was imaged using a 3.0 T MRI. The PA and MRI signal value of each group was measured.

### Statistical analysis

All quantitative data were presented as means ± standard deviation and was analyzed by SPSS 13.0 analysis software. One-way ANOVA was used to evaluate differences between multiple groups. *P*<0.05 was considered statistically significant.

## Results

### Characterization and identification of TSCs

**Fig 1:**
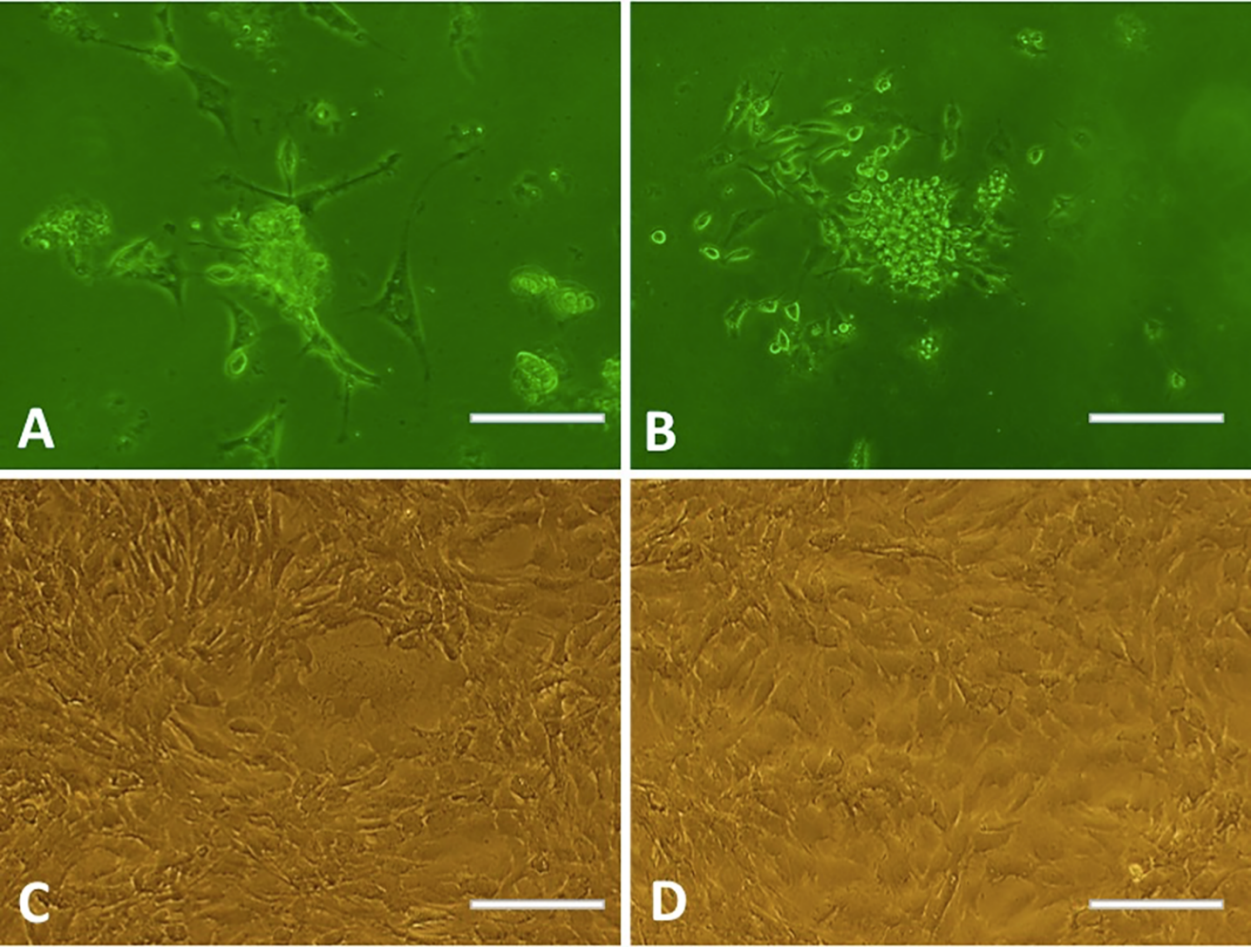
The clone and morphologic observation of TSCs by microscope. (A) P0 TSCs showed as large polygonal and star-shaped cells. (B) P1 TSCs showed as flat cells and slender fibroblast-like cells. (C) P3 TSCs showed a homogeneous population of fibroblast-like cells. (D) P3 TSCs arranged as pebbles at high rate of cell fusion. All scale bars = 100 μm.

**Fig 2:**
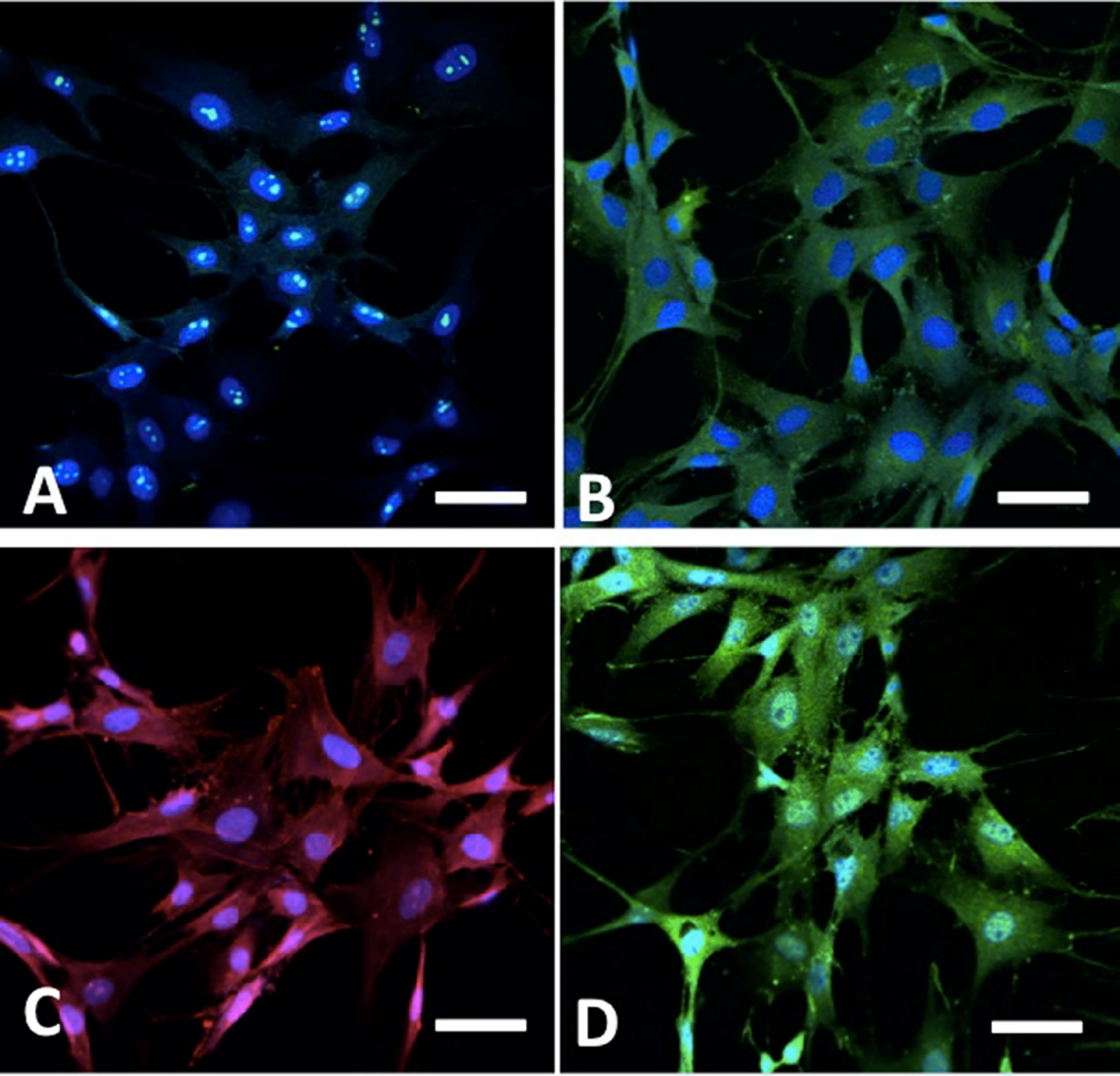
Immunofluorescent staining of Nucleostemin (A, green), CD44 (B, green), SSEA4 (C, red) and CD90 (D, green) in P3 TSCs. All scale bars = 25 μm.

To obtain TSCs for cell culture experiments, we first isolated the cells from rat Achilles tendons, observed their morphology and verified their stemness using immuno-staining. As shown in Fig 1, the P0 TSCs isolated from achilles tendon of rat attached onto the plate, and the colonies formed from single cells were observed at 7 (P0, Fig 1A) and 12 days (P1, Fig 1B). At P0, P1, and P3, large polygonal and star-shaped cells (Fig 1A), flat cells and slender fibroblast-like cells (Fig 1B), and a homogeneous population of fibroblast-like cells (Fig 1C) were observed. At a high rate of cell fusion, TSCs were arranged in samples characteristic of pebbles (Fig 1D). To identify that the TSCs were stem cells, we examined expression of TSC surface markers on the P3 cells using immunocytochemical staining. Our results showed that all TSCs were positive for CD44, CD90, SSEA4, and Nucleostemin (Fig 2).

### Fabrication and characterization of PLGA/IO MPs

**Fig 3:**
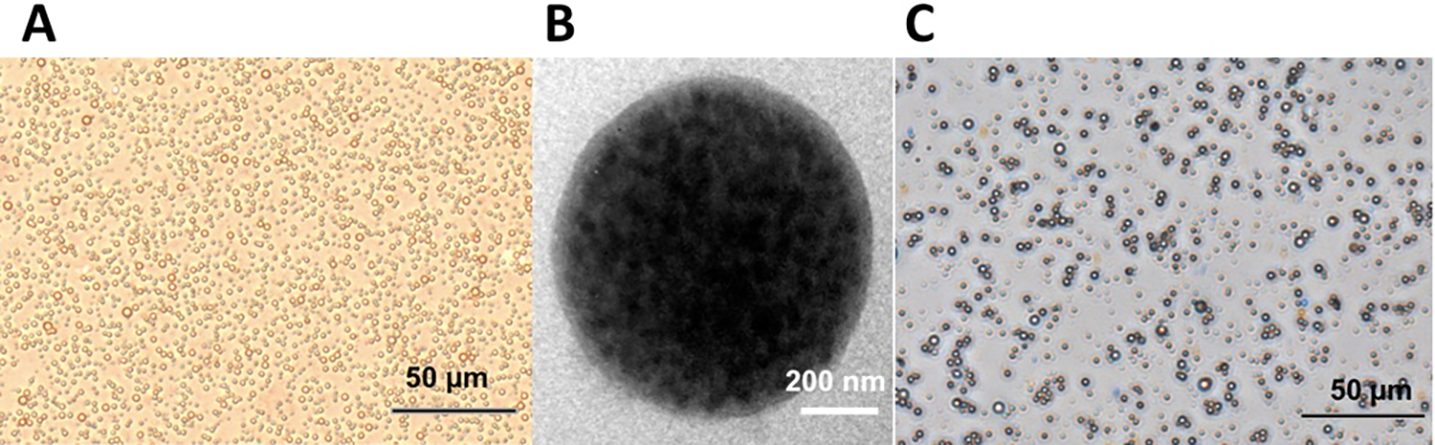
Characterization of PLGA/IO MPs. (A) Photomicrograph showed uniform size and even distribution instantly after preparation. (B) TEM image showed a spherical morphology with IO NPs encapsulated in the core. (C) Photomicrograph of PLGA/IO MPs after being stored for a month demonstrated no obvious aggregation or agglomeration.

PLGA/IO MPs were successfully synthesized using double emulsion method and demonstrated uniform size, good monodispersion, and even distribution by light microscope (Fig 3A). The average particle size and average zeta potential of the PLGA/IO MPs were 801.5 ± 165.6 nm and -6.36 ± 3.36 mV respectively (S1 Fig). Transmission electron microscope images (TEM) showed that PLGA/IO MPs were spherical in shape and IO nanoparticles were encapsulated within the core of PLGA MPs (Fig 3B), which indicated the successful loading of iron oxide. Any aggregation or agglomeration of the prepared PLGA/IO MPs were not observed after being stored at 4°C for a month (Fig 3C). The amount of Fe loading was 1.6 mg/mL in 1.5 mL PLGA/IO MPs solution using ICP.

**Fig 4:**
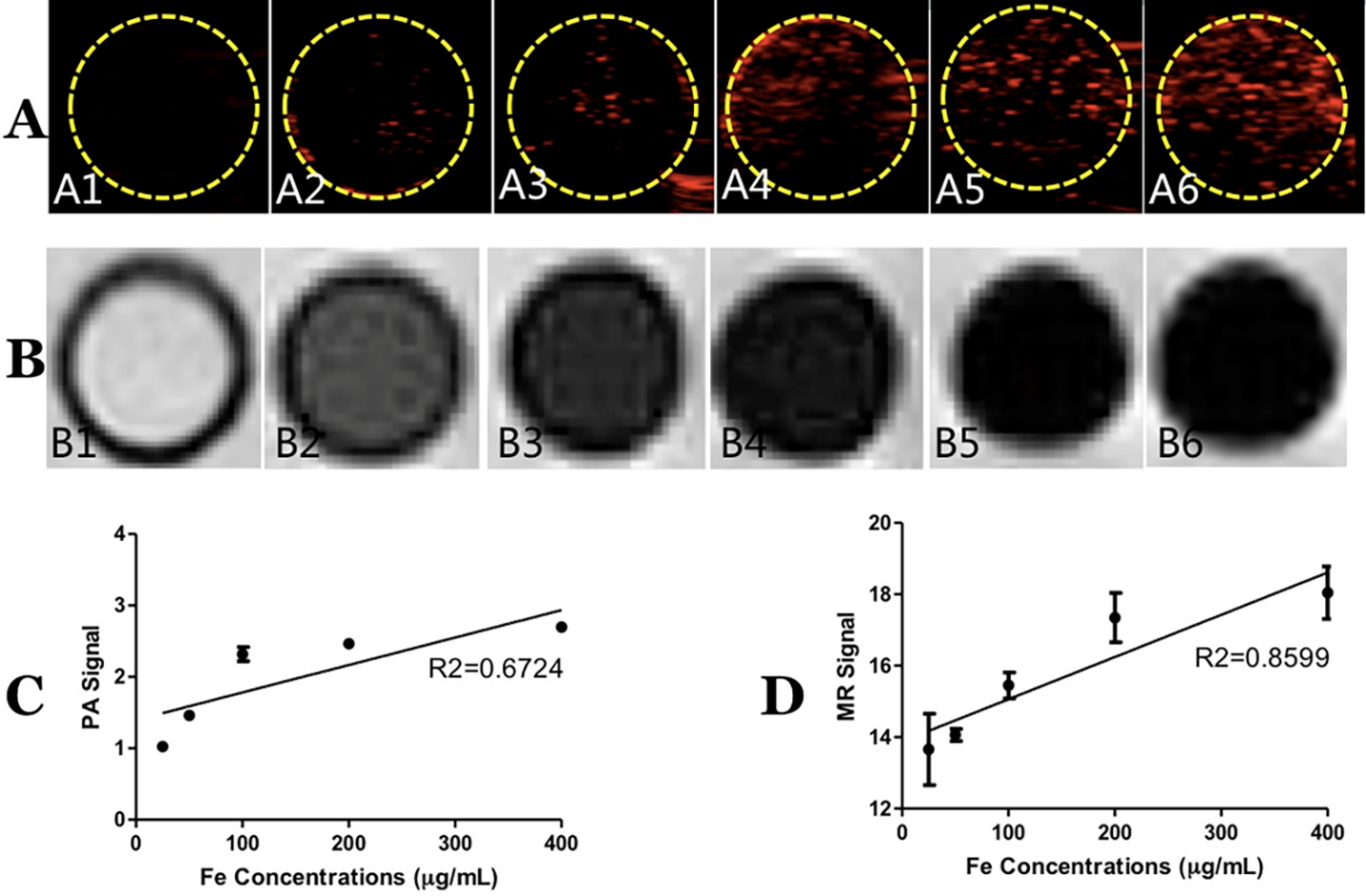
Dual-modal PA/MRI imaging of PLGA/IO MPs. (A) PA images of PLGA/H2O MPs (A1) and PLGA/IO MPs at iron concentrations of 25 (A2), 50 (A3), 100 (A4), 200 (A5) and 400 (A6) μg Fe/mL. (B) T2* images of PLGA/H2O MPs (B1) and PLGA/IO MPs at iron concentrations of 25 (B2), 50 (B3), 100 (B4), 200 (B5) and 400 (B6) μg Fe/mL. (C) The PA signal average value of PLGA/IO MPs at different iron concentrations. (*, *P* < 0.05). (D) The MR signal average value of PLGA/IO MPs at different iron concentrations.

PLGA/IO MPs at different iron concentration were subjected to both PA and MRI scanning in vitro with PLGA/H_2_O MPs as the control group to detect the property of PLGA/IO MPs for dual-modal PA/MRI imaging. As shown in Fig 4A and 4B, PLGA/IO MPs generated a positive PA signal (Fig 4A, the red in the yellow dotted circle) and a hypointense signal on T2* sequence (Fig 4B) compared with the PLGA/H_2_O MPs. It showed an increased PA signal and hypointense signal on MRI with an increased concentration of iron in the particles by qualitative observation. Meanwhile, the quantitative analysis by measuring signal intensity of each sample at different iron concentration confirmed that both PA and MRI signal increased significantly with an increased concentration of 25, 50, 100, 200 and 400 μg Fe/mL in PLGA/IO MPs (All *P<0*.*05*, Fig 4C and 4D). The PLGA/IO MPs at iron concentration of 100, 200, and 400 μg Fe/mL generated the most simultaneously readable PA and MRI signal.

### Labeling TSCs with PLGA/IO MPs

**Fig 5:**
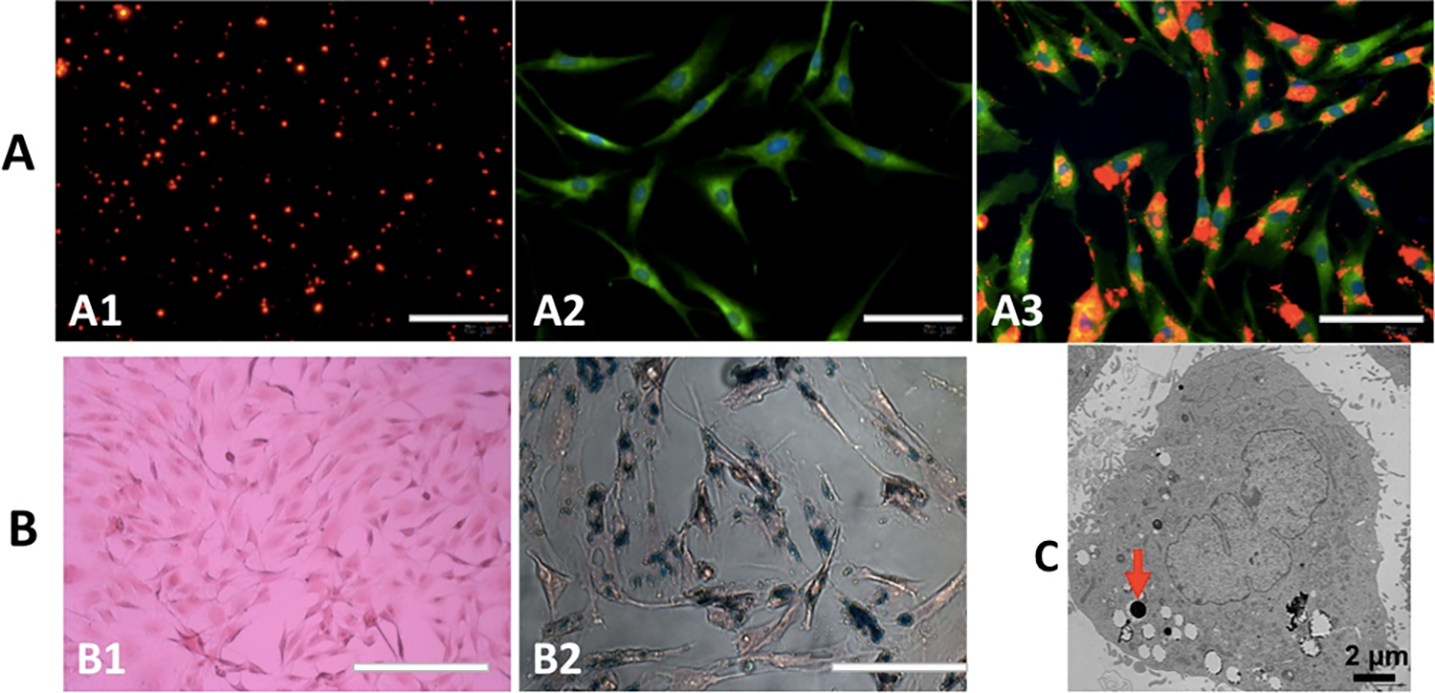
Identification of PLGA/IO MPs internalized within TSCs. (A) fluorescent staining of PLGA/IO MPs (A1, red), TSCs before (A2, cytoplasm as green, nuclear as blue) and after labeling with PLGA/IO MPs (A3, red particles aggregated in the cytoplasm). (B) Prussian blue staining of non-labeled cells (B1) and labeled cells (B2), blue-green staining PLGA/IO MPs were present in the cytoplasm, mostly around the nuclei). (C) TEM of labeled TSCs showed that high density PLGA/IO MPs (the arrow) were in the cytoplasm.

In the case of enhancing internalization of particles into the cell, the negatively charged PLGA/IO MPs (-6.36±3.36 mV in deionized water) were coated with poly-L-lysine coating leading to a positive surface charge of 3.16 ± 3.69 mV (S1 Fig). In a previous study described by Xu et al.[22], 100 μg Fe/mL iron concentration of fluorescent (DiI) PLGA/IO MPs was used to incubate TSCs for 12 h. As shown in Fig 5A, DiI-PLGA/IO MPs demonstrated as scattered red staining particles before incubation, aggregated in the cytoplasm around the nucleus after being encapsulated into TSCs. The Prussian blue staining also showed the blue staining of PLGA/IO MPs were aggregated in the cells (Fig 5B). However, we didn’t detect any particle within TSCs by fluorescent staining and Prussian blue staining in the control group (Fig 5A and 5B). TEM confirmed that the particles were present in intracellular compartments 12 hours after labeling (Fig 5C).

### Cytotoxicity of PLGA/IO MPs on TSCs

**Fig 6:**
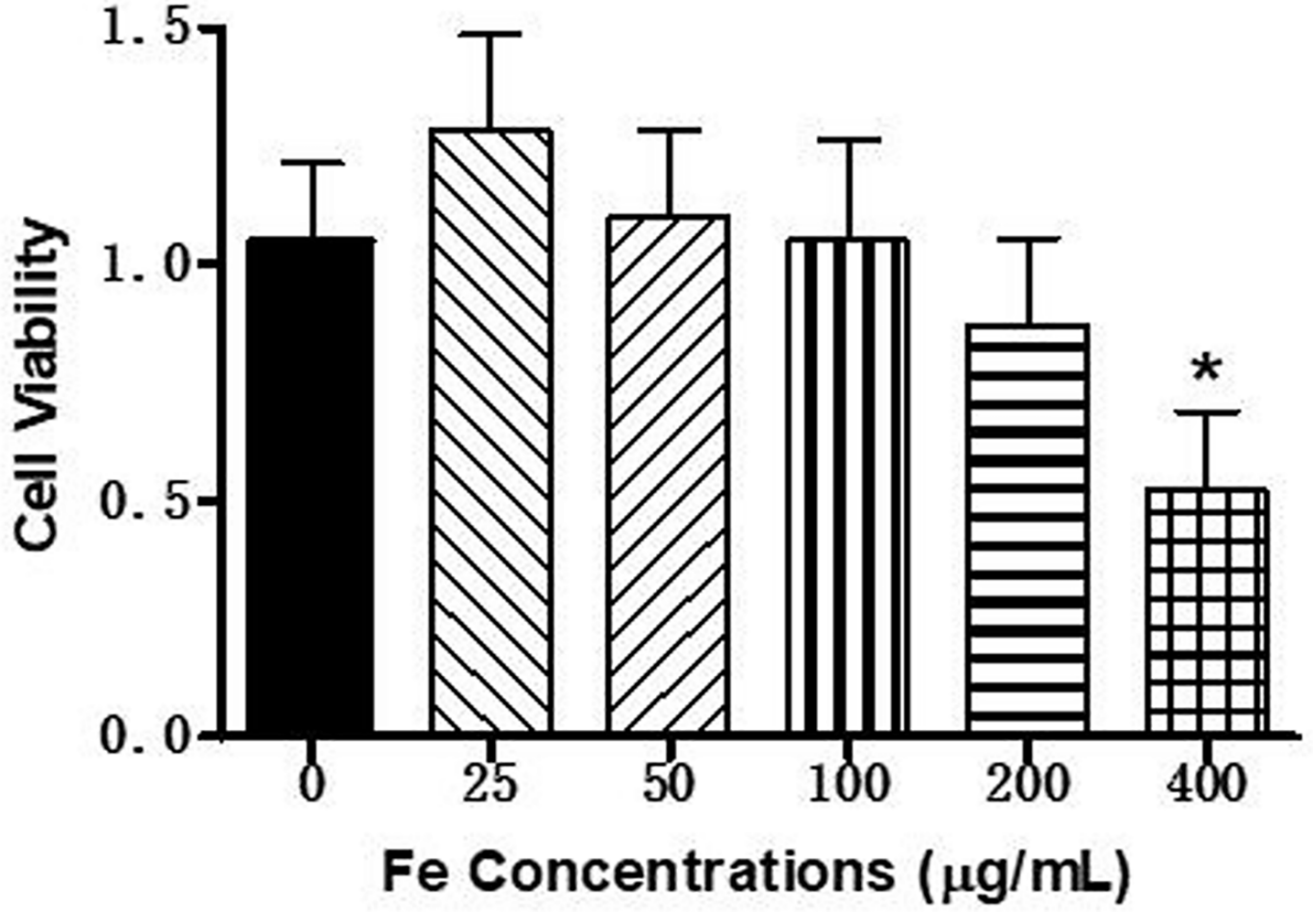
Impact of PLGA/IO MPs labeling on cell viability. * means *P* < 0.05 when compared with each other group.

In order to investigate the potential negative impact on the viability of TSCs, CCK-8 test was performed following PLGA/IO MPs internalization. As shown in Fig 6, there was no noticeable influence on cell viability for different iron concentration of PLGA/IO MPs at 25, 50, 100 and 200 μg Fe/mL compared to native cells 24 hours following particle internalization (all *P* > 0.05). The cell viability was significantly decreased in the group of 400 μg Fe/mL of particles compared to the group of 200 μg Fe/mL of particles for labeling (*P* < 0.05).

### Dual-modal PA/MRI imaging of TSCs labeled with PLGA/IO MPs

**Fig 7:**
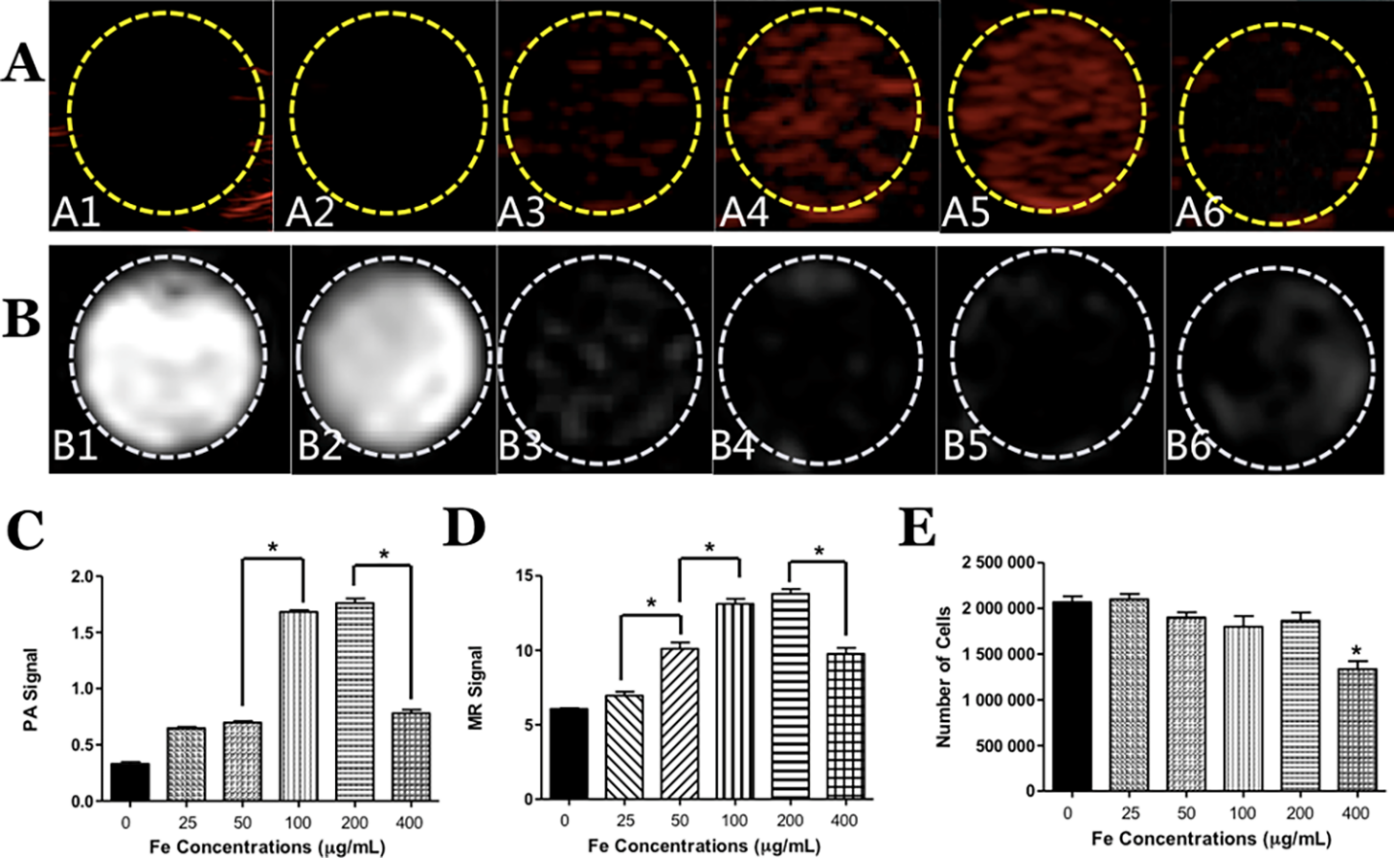
Dual-modal PA/MRI imaging of TSCs. (A) PA images of non-labeled TSCs (A1) and labeled TSCs with different iron concentration of PLGA/IO MPs (A2-A6: 25, 50, 100, 200, 400 μg Fe/mL in turn). (B) T2* images of non-labeled TSCs (high signal at B1) and labeled TSCs with different iron concentrations of PLGA/IO MPs (low signal at B2-B6: 25, 50, 100, 200, 400 μg Fe/mL in turn). (C) The quantitative analysis of PA signal, * means *P* <0.05. (D) The quantitative analysis of MR signal on T2* sequence, * means *P* <0.05. (E) Cell counting of TSCs in different group, * means P <0.05 when compared with each other group.

To evaluate the ability and sensitivity of PA and MRI for tracking TSCs simultaneously, TSCs were incubated with different iron concentration PLGA/IO MPs and imaged by PA and MRI. The non-labeled TSCs were utilized as the control group. As shown in Fig 7A and 7B, TSCs labeled with 50, 100, 200 and 400 μg Fe/mL particles demonstrated positive signal on PA images and low signal intensity on T2* images. While the non-labeled TSCs showed negative PA signal and high T2 signal, the labeled cells with 25 μg Fe/mL PLGA/IO MPs can barely be detected on both PA and MRI images. It indicated that the concentration of PLGA/IO MPs at 50, 100, 200 and 400 μg Fe/mL was applicable to label TSCs via both PA and MRI. As shown in Fig 7C and 7D, the quantitative analysis of average signal value revealed that both PA and MR signal increased significantly when the incubation of iron concentration increased from 25 μg Fe/mL to 100 μg Fe/mL. However, there was no obvious enhance of PA and MR signal in the group of 200 μg Fe/mL compared to that of 100 μg Fe/mL (1.76 ± 0.07 *VS* 1.68 ± 0.03, 13.80 ± 0.55 *VS* 13.12 ± 0.59, both *P* > 0.05). In addition, both PA and MR signal had a significant decrease at the iron concentration of 400 μg/mL compared to that of 200 μg/mL for incubation. Fig 7E showed the cell count of 2 well TSCs prepared for imaging in the group of 400 μg Fe/mL were significant lower than each other group, which may explain the decreased PA and MRI signal at 400 μg Fe/mL.

## Discussion

IO NPs demonstrated great potential as a multifunctional contrast agent used for multiple-modal imaging and the theranostics system. Some previous studies have suggested that SPION encapsulated PLGA NPs may be used as an alternative PA contrast agent[18]. Wu et.al had synthesized a NIR-active multidentate-polymers coated IO NPs and proved its ability for near-infrared fluorescence (NIRF)/PA/MR trimodal imaging of axillary lymph node[23]. The IO NPs mediated hyperthermic effect upon NIR-laser irradiation was also considered as a therapeutic agent for photothermal therapy and photodynamic therapy following targeted accumulation in tumors.

MRI cell tracking were realized by internalization of iron oxide materials into cells, thus the cells’ position can then be tracked by monitoring a decreased T2/T2* signal intensity at the anatomical site they accumulate. It has been determined in previous studies that larger particle size, positive surface charge, higher concentration, and longer labeling time are factors which can enhance the IO NPs endocytosis into cells, thereby improving the labeling efficiency[6]. Some coating or modification of IO NPs with cell penetrating peptides, dendrimers and polyamines such as poly-L-lysine (PLL), protamine-sulfate (PS), cationic lipid were reported facilitate the internalization of the nanoparticles[24, 25]. Xu et.al compared the TSCs labeling efficiency of micron-sized PLGA particles loaded with IO NPs (IO: PLGA-MPs, 0.8 um) and that of free IO NPs (10 nm core size), and concluded that TSCs internalization of IO: PLGA-MPs had an increased relaxivity, longer residence time inside the cells and higher R2 signal compared to free IO-NPs[22]. This demonstrated that encapsulation of IO NPs in a biodegradable PLGA MPs could be used as a potential strategy to improve cell labeling via MRI.

IO NPs have been clinically approved to track transplanted cells[26, 27] and have also been applied to label various kinds of cells like MSCs[28, 29], TSCs[30], and chondrocyte cells[31] via MRI. However, there have been little reports on IO NPs based particles used for dual-modal PA/MRI tracking of stem cell. In this study, we isolated and cultured TSCs from rat achilles tendon and detected the ability of PLGA/IO MPs for PA/MRI tracking of TSCs. PLGA/IO MPs were synthesized using double emulsion method, and showed good stability and dispersity without aggregation or agglomeration (Fig 3). Initially, PLGA/IO MPs were visibly detected by PA and MRI at the iron concentration of 50, 100, 200 and 400 μg Fe/mL. Simultaneously, the increased iron concentration of particles was also observed to result in greater signal intensity on PA and MRI (Fig 4). PLGA/IO MPs were then coated with PLL, and this resulted in the positive surface charge of particles which enhanced internalization of particles into the cells[22, 32]. Although both PLGA and iron oxide are nontoxic and good biocompatible materials that have been certificated with FDA approval, the prepared PLGA/IO MPs showed significant influence on cell viability at the high iron concentration of 400 μg Fe/mL following 12 hours’ incubation. The cytotoxicity may be caused by the occurrence of oxidative stress, which could be a cell response to a high load of particles or related to the generation of free iron[6]. Subsequently, we assessed the suitability of PLGA/IO MPs for in vitro tracking of TSCs via dual-modal PA/MRI modality. Those TSCs labeled with particles at iron concentration of 50, 100, 200 and 400 μg Fe/mL were detectable by PA/MRI. Contrastingly, TSCs labeled with 200 μg Fe/mL PLGA/IP MPs did not generate significantly greater PA and MRI signal intensity than TSCs labeled with 100 μg Fe/mL. It is reasonable to assume that the final Fe loading per TSCs in 200 μg Fe/mL group didn’t increase significantly compared to the 100 μg Fe/mL group. Bi et.al had also disclosed that the maximum Fe loading/cell was obtained at 100 μg Fe/mL particles for labeling, but further increases in the initial Fe concentration did not enhance the final quantity of Fe per cell[22]. PA and MRI signal intensity of TSCs in 400 μg Fe/mL group was significantly decreased compared to the 200 μg Fe/mL group. This may be related to the decreased total cell counts of labeled TSCs. Given that no statistically significant difference was found in PA/MR signals between 100 and 200 μg Fe/mL, to minimize use of reagents and potential cytotoxicity of particles, 100 μg Fe/mL was determined as the optical concentration of PLGA/IO MPs for labeling for further in vivo study.

The main limitation of this study was that we did not detect the labeling efficiency of TSCs of PLGA/IO MPs in vivo. Future study will focus on the non-invasive tracking of labeled TSCs in vivo by PA and MRI to determine long-term outcomes. Furthermore, histological, immunochemical, and biomechanical studies may establish the potential of the clinical application of TSCs for repairing injured tendons.

In conclusion, we found that PLGA/IO has the ability for dual-modal PA/MRI labeling of TSCs. Higher Fe concentrations of PLGA/IO MPs may also yield stronger PA/MRI signal of particles. However, high Fe concentrations may have detrimental effects on cell viability. Therefore, considering the balance between cell viability and PA/MRI tracking efficiency for labeling, we presume that good PA and MR signal of cell tracking without obvious cytotoxity to TSCs can be optimally achieved at 100 μg Fe/mL of PLGA/IO MPs concentrations in vitro.

## Supporting information

S1 Fig Size distribution and surface potential of PLGA/IO MPs. (A) Average particle size of PLGA/IO MPs was 801.5 ± 165.6 nm. (B) Average zeta potential of PLGA/IO MPs was -6.36 ± 3.36 mV. (C) Average zeta potential of the particles was changed into 3.16 ± 3.69 mV after being coated with poly-L-lysine (PLL).
(TIF)
