# Supplementary figures and images for: Dual-modal photoacoustic and magnetic resonance tracking of tendon stem cells with PLGA/iron oxide microparticles in vitro
Source: PLoS One. 2018 Apr 2;13(4):e0193362. doi: 10.1371/journal.pone.0193362 (PMC5880337; doi:10.1371/journal.pone.0193362)

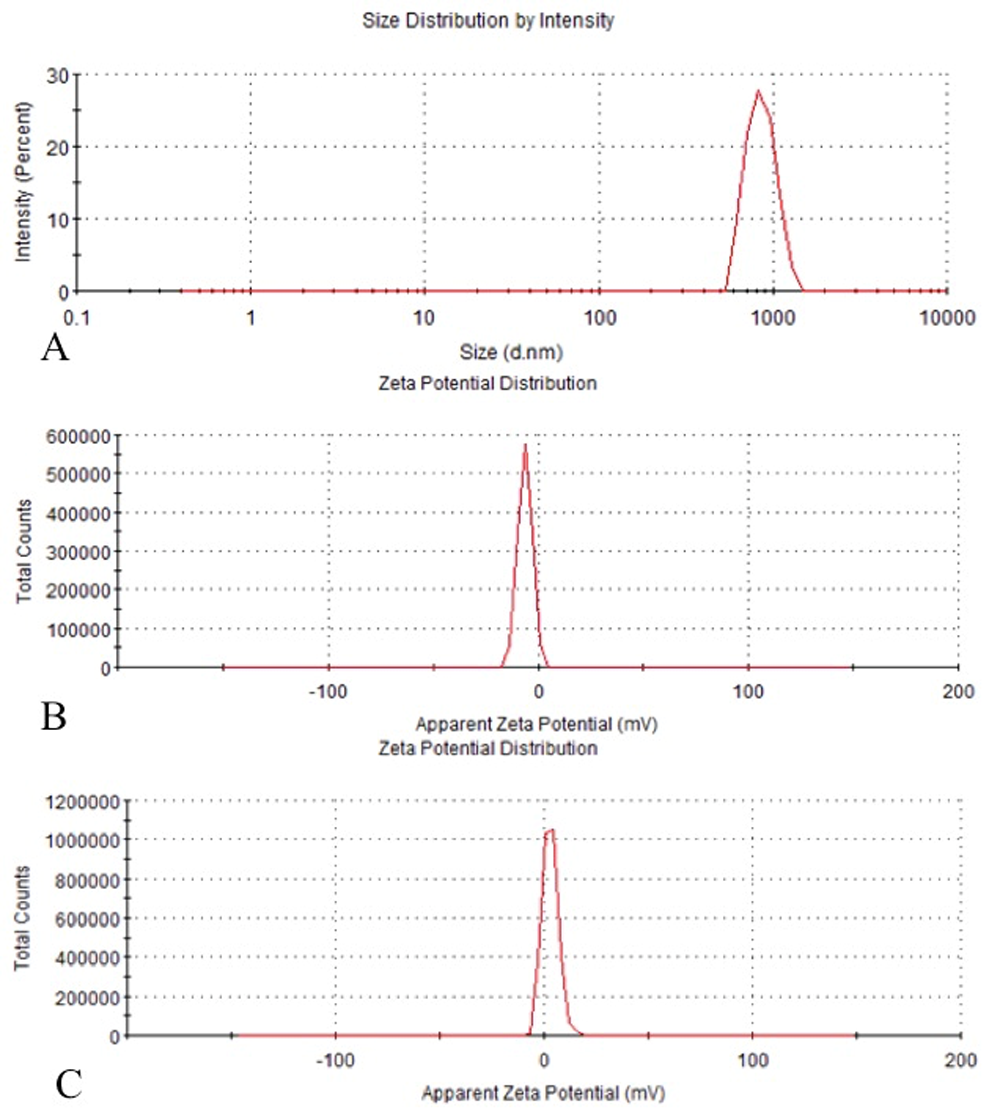

Supplement: S1 Fig — (A) Average particle size of PLGA/IO MPs was 801.5 ± 165.6 nm. (B) Average zeta potential of PLGA/IO MPs was -6.36 ± 3.36 mV. (C) Average zeta potential of the particles was changed into 3.16 ± 3.69 mV after being coated with poly-L-lysine (PLL). (TIF) [file pone.0193362.s001.tif]
